# Pain, Pseudoparalysis, and Periostitis: A Neonatal Presentation of Congenital Syphilis

**DOI:** 10.4269/ajtmh.25-0344

**Published:** 2025-09-25

**Authors:** Catalina Arango-Ferreira, Alvaro de Jesús Toro-Posada

**Affiliations:** ^1^ Universidad de Antioquia, Medellín, Antioquia, Colombia;; ^2^ Hospital San Vicente Fundación, Medellín, Antioquia, Colombia

After a home deliver, a 3-day-old, 36-week-gestation girl of indigenous origin was admitted with hypotonus, excessive crying, and paralysis of her right upper limb. The mother denied prenatal care and referred to herself being healthy during gestation period.

**Figure 1.**
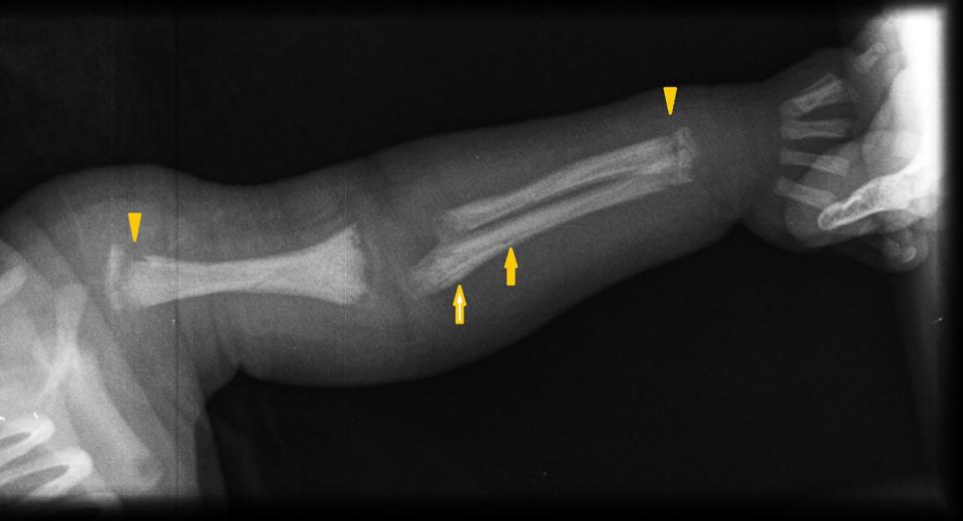
Radiographic evidence of periostitis and metaphyseal bands in upper left extremity long bones.

**Figure 2.**
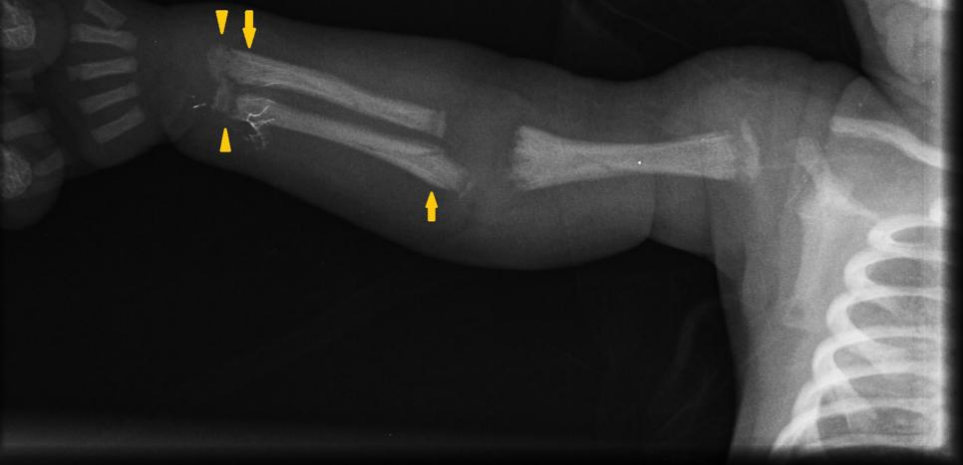
Radiographic evidence of periostitis and metaphyseal bands in upper right extremity long bones.

**Figure 3.**
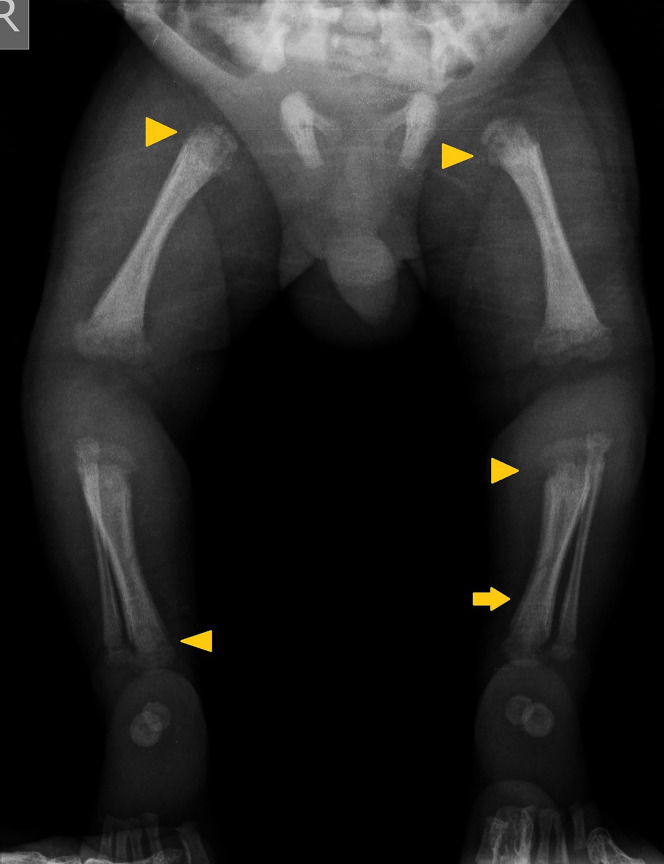
Radiographic evidence of periostitis and metaphyseal bands in lower extremity long bones.

Physical examination of the girl revealed fever (102.2°F), marked irritability, and crying upon manipulation. Gestational age was 36 weeks with low birth weight (2,300 grams). Intravenous ampicillin and gentamicin, considering neonatal sepsis, were initiated. Two blood cultures came back negative for aerobic bacteria. Labs included thrombocytopenia: 71,000/*µ*L, cholestasis (total bilirubin: 8.67 mg/dL, direct bilirubin: 6.45 mg/dL), and normal aminotransferases. Long bone radiographs ruled out fractures, and due to infant’s serum VDRL of 1:2048, showed generalized metaphyseal bands and periosteal reactions. (Figures 1, 2, and 3). CSF VDRL was positive.

Maternal HIV and Hepatitis B surface antigen were negative and serologic VDRL was 1:128.

Congenital syphilis with neurosyphilis and periostitis was confirmed and the infant received 10 days of aqueous crystalline penicillin.^1^ Resolution of the infant’s irritability, excessive crying, and complete mobility of the right arm compatible with Parrot’s pseudo paralysis (apparent paralysis of an infant’s limbs due to pain caused by syphilitic periostitis) were noted. Follow-up serum VDRL tests became negative.

Congenital syphilis is increasing worldwide.^1^^,^^2^ It can be difficult to confirm with compatible signs not always present. Placental transfer of maternal treponemal and non-treponemal antibodies can make tests difficult to interpret.^2^ Consideration of the mother’s gestational history for syphilis, prenatal care, a prompt diagnosis and treatment, and newborn examination are fundamental to prevent deafness, neurological, and other severe sequalae.^2^^,^^3^ Clinical signs highly suggestive of congenital disease with positive non-treponemal antibodies confirm diagnosis.^2^^–^^4^ Differential radiological diagnoses include scurvy and vitamin A deficiency.
